# The League of Mercy: Annual Meeting at St. James's Palace

**Published:** 1913-12-27

**Authors:** 


### THE LEAGUE OF MERCY.

### Annual Meeting at St. James's Palace.

The fourteenth annual meeting of Presidents of the League of Mercy was held at St. James's Palace on Thursday, December 18, his Serene Highness Prince Alexander of Teck, G.C.B., G.C.V.O., D.S.O., Grand President of the League, occupying the chair. Supporting him were H.R.H. Princess Alexander of Teck and the Presidents, Lady Presidents, and hon. secretaries of districts.

The minutes of the last annual meeting, held on December 16, 1912, were read and duly confirmed.

### Prince Alexander of Teck's Address.

His Serene Highness said it gave Princess Alexander and himself great pleasure to be present again at the annual meeting of Presidents and meet the honorary officers of the League of Mercy for the coming year. The apprehensions felt in many quarters last year that the working of the Insurance Act would militate against the voluntary hospitals had not been dispelled, although they had been somewhat lightened by another year's experience. The beneficent work of the voluntary hospitals was so well known, so knit up with our social life, and was so happily associated with the freedom of the medical profession and the needs of medical education, that it had continued to receive public support and to command public confidence. The uncertainties exhibited both here and in Germany, which appeared to be incidental to any scheme of nationalisation and State endowment of medical and surgical relief, had not tended to encourage an attempt to substitute State hospitals for voluntary ones. (Hear, hear.)

It was gratifying to know that King Edward's Hospital Fund was, for the third year in succession, distributing the same sum—namely, £157,500 from its usual sources of revenue—among the London hospitals or sanatoria and convalescent homes which were receiving patients. The League of Mercy continued to make awards to London hospitals within its area of collections, and outside the county of London awards to £2,318 were so made to such hospitals, the largest amount yet contributed to extra-Metropolitan institutions. Numerous entertainments were given for the League during the year, and on May 24 was held the admirable concert at the Albert Hall, under the patronage of the King and Queen, in the presence of the Prince of Wales, Princess Mary, and many other members of the Royal Family. All active workers for the League were invited, and although profit was not sought, over a hundred pounds were handed over to the Samaritan Fund, while contributions were made to the Press and to the Actors' Benevolent Fund. For the great success and pleasure which the concert afforded their thanks were due to Sir James Harrison and Mr. Ronald, who so kindly gave their services, and Lady Alexander. They had to regret the loss by death during the year of Lady Strathcona, the Lady President for Hitchin, Mrs. Leicester Keppel, Lady President for Worthing, and the Rev. E. P. Anderson, Vice-President for South Kensington. If one were to make the unjustifiable and impossible assumption that each district within the League's operations was fully at work, and that every officer and member were able to achieve the full complement of success, there should have been a revenue this year of £100,000. (Hear, hear.) This, of course, was assuming the impossible; it was a counsel of perfection. Owing to many adverse causes it was a difficult question to attain, even in the best worked districts, the maximum amounts. In the year 1912 only two districts reached such maximum, while five districts secured over 50 per cent., and some sixteen districts secured over 25 per cent., as judged by this well-nigh unattainable standard. If all the districts were able to secure 25 per cent. of the standard, the League would be actually enjoying an annual income of £27,000, whereas the highest revenue, reached in 1908, was £21,660. He was not suggesting any reflection or criticism on their arduous and difficult labours, and he realised the varying conditions as regarded the wealth of the population, distance from London, etc., obtaining in the ninety districts; but it appeared to him that in the interests of the League, and the objects they had at heart, it might be well for the Executive Council to consider whether in those districts which over a series of years showed less than 10 per cent. of the standard there should not be a reorganisation effected, and possibly some change made in the presidencies or vice-presidencies therein. Before resuming his seat he would like to make mention of the eminent services rendered to the League by its honorary officers.

It would be invidious to make distinctions where all had worked so willingly and so well; but he wished specially to refer to the Honorary Treasurer and Honorary Secretaries. Sir Henry Burdett might fairly be termed the originator of the League, and it was largely owing to his expert knowledge in regard to charitable institutions that this auxiliary to King Edward's Hospital Fund came into vogue. Sir William Collins and Sir James Harrison had been associated with Sir Henry Burdett in this good work since its inception, and it would be impossible to exaggerate the valuable services they had both rendered. And he was glad to be able to inform the assembled company that the King had been pleased to approve of the honorary officers receiving the Order this year. (Applause.) In conclusion, as Grand President and on behalf of the Lady Grand President, he desired to offer to all present and those associated with them hearty congratulations on the excellent work they had done for the League, and to say, Still better next year. (Applause.)

### The Right Hon. Lord Clarendon moved: "That the Right Hon. the Lord Farquhar, G.C.V.O., be appointed Chairman; Sir Frederick Green, J.P., Deputy-Chairman; and Captain A. E. Speer, Hon. Secretary, respectively." The close, and even intimate, friendship with which Lord Farquhar was honoured by two successive Sovereigns, and Lord Farquhar's well-known sympathy in support of many beneficent and charitable institutions, the conspicuously successful manner in which he had administered the affairs of the Order, gave him, in Lord Clarendon's opinion, a prescriptive right to the post to which he had the honour to propose him. And the same remark applied to the other two gentlemen named in the resolution. They were fellow-labourers with Lord Farquhar in the same vineyard, and, though he presumed they would not receive the orthodox penny, they would receive a much higher and nobler reward—namely, the appreciation and gratitude of all with whom the League was brought into contact.

**Mr. W. J. Chamberlain**

seconded. It would be a work of supererogation to add to the sentiments expressed by the proposer, and he would content himself with the remark that actions spoke louder than words. If this meeting should be pleased to elect the gentlemen to the posts concerned that would be the most effective way of showing appreciation for their work in the past, and an expression of confidence for the future.

Carried unanimously.

**The Viscount Hill**

proposed: "That seven representative members of the Executive Committee be appointed: The Right. Hon. the Lord Farquhar, G.C.V.O., Col. H. B. Hamilton, Sir Frederick Green, J.P., Mrs. R. L. Harrison, Mr. Edward Murray Ind, D.L., J.P., Lady Dimsdale, and Sir George Alexander, LL.D., J.P." These ladies and gentlemen were, he said, so well known that he thought it was quite right to accord them a place on the Executive.

**Colonel B. T. L. Thomson, V.D.**

seconded, and endorsed what Viscount Hill had said. This was the Committee upon whom rested the real responsibility for the work of the League. In these times, as the Grand President had said, it was a difficult work to collect subscriptions, but he hoped something could be done to increase the funds next year.

Carried.

H.R.H. Princess Alexander of Teck then presented the Order.

**Sir Henry Burdett**

said he wanted his hearers to get clearly into their minds the fact that the cause of any falling off in 1913 was quite definable and entirely special. Most workers began the year in a terror of National Insurance. National Insurance had dogged their footsteps all through the year, and when workers were discouraged at the beginning the results at the end were usually not entirely satisfactory. It was a matter for great regret to him, as he was sure it would be to those present, that two of the most energetic, successful, and precious workers of the League were unable, through illness, to be present on this occasion. Dora Lady Chesterfield asked him to convey to her Royal Highness and his Serene Highness her regret that she was unable to attend the meeting, on account of having met with an accident. Lady Chesterfield had never failed to be present before, and although she could not be present in person she was with them in spirit. (Hear, hear.) He also had to express sincere regret, which everyone would share, that Miss Enthoven, the Hon. Secretary of the Paddington Branch, whose energy and organisation were well known, was unable to attend, as her father died suddenly the previous morning. The position, as he had already said, was this year depressing; but, after all, a recent prize fight seemed to show why the League was not in the position it was last year. If one entered a fight feeling one might be beaten the probability was that one would be beaten, and the beating would be deserved. In the course of his speech he hoped to show that all the causes which had been dreaded were behind them, and that they might go forward next year with increased vigour, and certainly with assured success.

The Results in 1913.

During the past year there had been ninety-two districts at work, as against ninety-one in the previous year, and if one took the average of all the districts the falling-off represented, in those which showed a falling-off, not more than 10 per cent. That 10 per cent. could be easily recovered, and the League could go forward to much larger collections if the necessary energy were put into it. But on the other side was the pleasant fact that, despite all the discouragements and fears, thirty-five districts showed an increase in their contributions this year. For instance, Fulham showed an increase of £58. Fulham was the first of all the districts for many years, a proud position due to the former President and to Mrs. Harrison, who had been one of the most zealous workers in the League, and was still a tower of strength. Fortunately for themselves there was a new President for Fulham—namely, Mr. Philip Walker—who, he understood from Mrs. Harrison, was the cause of Fulham forging ahead. He looked forward with confidence to the period when Fulham would resume its proud place at the head of the collections. Esher showed an increase of £73, Berkshire £11, East Sussex £8, and Greenwich £91.

The increase at Greenwich was owing to the work which had been done in that district, where Lord Hill, Mr. Stone, and Miss Stone had shown commendable energy, which deserved success. Oxford showed an increase of £20. That was very important, because at the beginning of the year he received a very earnest letter from one of the workers there, Lady Dillon, who said it was hopeless to expect to continue to raise money in an agricultural district like Oxford, owing to the Insurance Act. She begged that he (Sir Henry) would go and speak to the workers. He had pleasure in acceding to that request, and he put before the people the fact that so far as National Insurance was concerned if it had any effect upon their attitude and work for the League, the effect should be to increase their efforts for the hospitals, and this for several reasons. One was that the strength of National Insurance in this country at the present time, financially speaking, rested upon the free in-patient relief which was being given to insured persons by the voluntary hospitals. The League's work was all for the voluntary hospitals, and therefore everybody who needed a hospital and everybody who took an interest in hospitals should certainly be joining the League of Mercy and giving personal service in the cause of the sick in order to help those poor people to get what they lacked. The effect of that was that all had rejoined, and the result as expressed in figures was a unique and encouraging departure in the case of Oxford—namely, £20 increase in this bad year. He commended that fact to the reflection of those who felt depressed; he mentioned it as an example which should be followed. Hornsey and Finchley showed an increase of £95, the largest increase in the year. Sevenoaks showed £3, Epping £12, Eastbourne £7, Maldon £5, Brighton £43, Reigate £17, Woolwich £10, Medway £13, Ealing £10, Hitchin £6, Wimbledon £18, Romford £1, Enfield £22, Hackney, Central, £33, Watford £2, St. Pancras £41. St. Pancras was a new district, and £50 was collected in a short time by the new Lady President, Mrs. H. H. Clutton, who had done good service for the League for many years. Lewes showed an increase of £6, Camberwell £11, and this was chiefly due to the energy of Miss Meadows, the hon. secretary. Holborn showed an increase of £30 owing to the energy of Dr. Kennard and Mrs. Kennard. Holborn had been very difficult to work, and it was an encouraging fact that the increase should have been won in the worst year. Colchester showed an increase of £5, Poplar and Limehouse of £4, and there were increases also at Sudbury, Harwich, and Faversham. In the Isle of Thanet the increase was £3, and he would draw especial attention to the fact that in Bethnal Green, the district of so many poor people, there was an increase in this bad year of £13. (Applause.) Against the district of Chichester appeared the words, "Begins to work." He knew that district, and the fact of its getting to work for the League of Mercy meant that the League was rapidly rising in popularity.

The approximate total for the year reached £17,227. That, of course, showed a falling-off compared with last year, but last year a large contribution was received from the Albert Hall concert. There was, he was grateful to say, a further contribution specially sent in anonymously by a few people who took a great interest in the League, in response to his request, made in that room, that there should be forthcoming a spontaneous extra gift of £1,000, so as to make the total contributions to hospitals reach £200,000. And here he wished particularly to refer to the splendid services being rendered by those whom they were glad to look up to as leaders. In the case of Croydon her Royal Highness the Duchess of Albany had done something which was really inspiring. She already had three districts to look after, but as Croydon, which used to give a good yield, had languished for some years, her Royal Highness had kindly stepped into the breach and with great courage became the President there. (Hear, hear.) In the result Croydon was being thoroughly well organised, and her Royal Highness had the help of Mr. Chamberlain, who had already spoken that day. Mr. Chamberlain was superintending the reorganisation of the district with a view to increasing the collections. Sir Henry would say of Croydon that in the course of the inspections which he had been making of voluntary hospitals up and down the country he found himself in Croydon about two months ago, and spent some pleasant hours in that hospital. It was a most excellently managed institution, and had the brightest and most delightful ward for children to be found in the hospitals of this country. That was a good omen, and if Croydon did not wake up under Mr. Chamberlain's presidency, with the encouragement and presence of H.R.H. the Duchess of Albany, he should be very much disappointed. He wished also to mention that H.H. Princess Victoria of Schleswig-Holstein, who had four counties to look after—Berkshire, Buckinghamshire, Hampshire, and Oxfordshire—was able to show good work under her energetic guidance, for more than one of the districts in those counties had secured an increase in this bad year. Their Royal Highnesses had good cause for their faith in the League. It had already made grants this year to extra-Metropolitan hospitals amounting to £2,318, the largest annual sum which the League had yet given away to hospitals outside London. The League had altogether contributed to the King's Fund £201,034, making with the £14,383 given to extra-Metropolitan hospitals since the beginning of the League a total sum of £215,413, raised mainly in small subscriptions of one penny and upwards, during a period of fourteen years. That result should cause every member of the League to thank God and take courage.

### How to Raise £50,000.

The League of Mercy was neither dead nor dying. Those who thought it was, or who were tiring of the work, he was sorry for, but the facts did not support their views. They believed the future of the League of Mercy was going to be increasingly fruitful in results. They were only at the beginning, because they had not yet raised half the sum which King Edward hoped would be collected—namely, £50,000 a year in small contributions. In this connection he did not think it would be his duty to hold out a hopeful prospect without telling those present, as a practical man of business, where he thought districts sometimes failed. He believed they aimed too high; they had been going too frequently to the people who gave guineas and larger sums. The League of Mercy was always intended for the humble folk. That he was right in urging this was proved by the facts that a district like Bethnal Green had yielded an increase of £13 this year, and several other districts which showed an increase were largely inhabited by the working classes. Wherever there were working-class communities the League had progressed and showed an upward tendency. His experience convinced him that the people to approach and thoroughly attack were those in the shops, the offices, the factories, and workshops everywhere. Again, there were butlers, who contributed to the League of Mercy £70 or £80, and one butler brought him over £100. The butler was a very important person. Among the workers of the League were many sportsmen. So he suggested that at this season of the year the head gamekeeper might be worked with success. (Laughter and applause.) If a gamekeeper took a card, it would not hurt him to contribute £10 a year. Amongst others who had helped very much were the postmen, the policemen, the flower-sellers, and the rag-and-bone dealers and collectors, some of whom did very good work. He (Sir Henry) was now getting an old man, and probably he would not be there to speak many more years; but the longer he lived the more grateful he felt that he had had the privilege of advocating in this country the doctrine and the principle of personal service in the days of health in the cause of the sick. There was nothing which anyone, however humble or however great, could do which would bring to them, in their hours of trial and on the sick bed, more comfort than the knowledge that in their strength and working days they had devoted time and themselves to spontaneous acts of personal service for the sick. He heard only the other day from the nephew of a lady, a vice-president, who had done most wonderful service and raised over £1,000 for the League, that when she lay dying in her room, where her certificate of the League of Mercy hung on the wall, she found comfort in the remembrance that whilst she was well she had done what she could. That was the principle upon which the League rested, and in this spirit it must go forward.

### Roads to Success.

He wanted to impress the usefulness of selecting popular workers and appointing each one a captain of five streets, so that all the streets in each district of the Metropolis where there was a branch of the League might hear of its objects and service. Each captain of five streets to undertake to call once a month for the pennies at each of the houses, and where that principle had been established in connection with hospitals in distant parts of the country, the yield had been most encouraging. Last year he asked for a spontaneous gift of a thousand pounds, which was sent to him, and he felt very grateful. This year he asked, not for money, but for gifts of personal service. He wanted, as Treasurer, a Body Guard of six hundred men and women to send their names and addresses to him by January 1, 1914, with an offer to join the League and work for it under his direction for one year. When the names reached him, he would assemble the Body Guard and go forward with the movement. If these names could be posted to him he was confident that when he met them and explained the whole matter to them, and they returned to the various districts in which they resided and worked for the League, they would find that the result of the efforts of these six hundred people would astonish London, and cheer the hospitals and the workers, too.

PROOF BY RESULTS.

He was emboldened to make this invitation because he had before him the result of the efforts of one of the Vice-Presidents, who had been working for the League twelve years. She sent in £80, and stated that of that £80, £60 had been collected in pennies; the penny collecting cards—one penny a week or a month—had been one of the most active and successful of the planks on which she rested her work. The pennies were always given willingly, and were ready when called for. Moreover, the sum in each case was such that it was not missed, and the service it invited was fruitful in blessing. Therefore, he would say to the Presidents of the League, Go lower and spread your net wider, and your success may astonish you.

THE TRIUMPH OF THE VOLUNTARY SYSTEM.

He assured his hearers that the voluntary principle, like the principle of personal service, was very much alive. Personal service now claimed a club—the "Cavendish Club"—and there was a Personal Service Association, the idea of which the Bishop of London had with great wisdom taken from the League of Mercy, which was very successful. The voluntary principle was stronger in the hearts of the people of this country to-day than ever before.

It was true it had been threatened by National Insurance, in the view of many people, but it had survived that crisis. Sixteen years ago King Edward's Fund was started, and in the period since that Fund had raised and distributed among hospitals and convalescent institutions one and three-quarter millions of pounds, and it had besides £1,800,000 of invested funds. All agreed its influence for good, as a centre for all kinds of hospital effort in the Metropolis, was tremendous. But there was one other thing, even more important than that, proving that the voluntary principle was not dead. If one examined the facts and figures of those sixteen years, and took the actual work and the cost of the work of all the voluntary hospitals of this country, the results were these. That whereas, owing to the enormous increase in the cost of treatment, and under other heads too, the expenditure of the voluntary hospitals had continued to increase year by year, until the total cost in 1912, compared with that in 1897, exhibited an increase of 70 per cent., during these same sixteen years the actual increase in money which the hospitals received in voluntary gifts amounted to a sum equal to 66 per cent. of the income in 1897, so that the actual deficiency was but 4 per cent., despite the huge growth in expenditure. He commended these figures to the attention of everyone who was fond of decrying the voluntary principle, and who thought it was dead. It really was never so much alive as to-day. It was now stronger and better, because the younger generation were beginning to realise that their welfare, the good of this country, and the cure, protection, and care of the poor in sickness, owing to the voluntary system, rested on the safest foundation. This system was the safeguard of freedom for the medical profession, of the advancement of medical education, and of the safety and protection of the patients. So it rested upon the securest foundations that the world had yet provided for hospital treatment.

**Sir William Job Collins**

said he did not think it necessary to add, as Secretary, much to the words which had fallen from the President and from the Honorary Treasurer. He would merely wish to say, on behalf of the honorary officers, that they gratefully acknowledged the gracious words which fell from the President in the earlier part of the proceedings in recognising the services which they endeavoured to perform on behalf of the League. He thought there were a few points in which it was well to remember the King's Hospital Fund, of which this League was a valuable auxiliary, differed from the League. In the first place the King's Hospital Fund was increasingly deriving its funds, in larger and larger proportion, from invested revenue, whereas the League of Mercy every year had to collect the whole amount which it distributed. In the second place, the King's Hospital Fund made its distributions wholly within the County of London, whereas the League of Mercy made donations also within the area of its operations, namely, the home counties as well. Those facts should be borne in mind when comparisons were made between that great body and this League. On behalf of the Executive Council he wished to say that they were always ready, as now, to receive suggestions from Presidents and Vice-Presidents or members which might be valuable to increase the efficiency and work of the League, and he had no doubt that the suggestions which fell from the President in the opening remarks would tend to speed up the machinery of the League and be productive of a more satisfactory report as a result of the appeals next year.

He would add with regard to the Insurance Act, which had been very frequently alleged as a reason for the falling off in the year's receipts, that at any rate one fact clearly emerged, namely, that after another year's experience of the working of the Act, the need for relief given to the sick by the London hospitals, and therefore the demand which they still had to make upon the charitable public, would come out as clearly evident—*i.e.*, the London hospitals during the past year had continued to relieve some 2,000,000 sick insured persons in London, to keep available 10,000 beds, which were constantly filled, and to spend some two millions of money in carrying on their beneficent work. (Hear, hear.) It was clear that their voluntary agencies would not be superseded by the operations of the Insurance Act. Indeed, the authors of that Act disclaimed from the first any intention to interfere with the working of the voluntary hospitals; in fact, it was authoritatively stated, so anxious were they not to interfere, that no provision was made of a definite character to subsidise them lest it should be a means of interfering with that voluntary principle upon which the authors of the Act set so much store. It was true that the position was somewhat unstable at the present time. If an illness were trivial or an injury slight, it was deemed to be the affair of the panel doctor and the State; whereas if the injury were severe or the disease serious and grave, it was not the affair of the State but of a voluntary charity. That was partly the reason why persons were still somewhat apprehensive as to how the National Insurance scheme would work out eventually. Some thought that under a certain power given in the Insurance Act, Insurance Committees or

Approved Societies would subscribe to the funds of the voluntary hospitals. So that one might hear to what extent that was taking place, a letter was addressed by the Secretary of the League to the Insurance Commissioners, and the reply ran : " In reply to your letter of the 21st inst., I am directed by the National Health Insurance Commissioners to state that they regret they are not in a position to furnish you with the information requested therein." From *viva voce* interrogation he had ascertained it was not because the amounts had been so considerable or incalculable, but that they did not exist. As a matter of fact, the sick benefit which under the original Act was by some hospitals appropriated for the institution, under the amending Act of this year could not be devoted to that purpose, but must be returned to the insured person in cash or in kind. It was asked how it was. If there was a compulsory contribution on the part of the employer and employed, and when the insured person was treated not by a panel doctor but by voluntary hospitals, from whom should the contributions come? That had not yet been answered. One heard of £140,000 existing in London in a suspense account on behalf of the Insurance Committee because people had not, either from the robustness of their health or from indifference towards the panel doctor, put themselves upon the list. Therefore this sum of £140,000 remained unallotted. He did not suggest to whom this money equitably belonged; but if there was a difficulty in disposing of the amount, the London hospitals would be able to put it to very useful purpose. He would like to emphasise the remarks which fell from the President as to the importance, not only of the voluntary system as such, but because it carried with it that freedom for the medical profession and for medical education without which a liberal profession could not possibly make advance in knowledge, or institute those free investigations which in their usefulness would be a source of so much beneficent activity, and which would flourish under a system which was free and not enclosed in a straight waistcoat. Any thanks which the League were good enough to bestow upon the officers must be handed on to those who, at the Central Office, daily carried on the labours of the organisation, especially Colonel Kempster, who was known to all, and his assistant Miss Milnes, who was always ready to help.

Father Bernard Vaughan then delivered an address, a full report of which appears on page 335.

### Alderman Sir Charles Wakefield, L.D., J.P., proposed, "That the Rev. Father Vaughan, S.J., be thanked for his kindness in giving his address." He was sure the meeting would wish to give a very hearty vote of thanks to Father Vaughan for his soul-stirring address. His eloquent words must have gripped the hearts of his hearers and their imaginations, and they would be a stimulus to the League's workers. Father Vaughan had offered to lecture on behalf of the League, and he was sure that kind offer would be gratefully accepted.

### Captain Monteagle Browne seconded the resolution with the greatest pleasure, and congratulated the committee on their wisdom in asking the learned father to give the address. A very distinguished member of the Church of England, the Bishop of London, gave the address last year; and as a worker in the East End of London, he asked whether it would not be well to ask the Jewish Chief Rabbi to perform the same function next year, to show that the League of Mercy was quite above all creed; and he had seen creeds swept away in the kindly co-operation on behalf of some such institution as the League of Mercy. By always being ready to help and comfort those who fell by the way, the best would be done to further the highest patriotism and in opposition to that wave of Socialism which, if the people were not mingled with, might sweep this country to destruction. Father Vaughan's work in the East End, which was honeycombed with philanthropic institutions, did him the highest credit. He hoped the labours of the League would be so spread that the purse of the land would be gripped.

Carried by acclamation.

### The Lord Farquhar, G.C.V.O., proposed a cordial vote of thanks to his Serene Highness Prince Alexander of Teck for the able way in which he had presided over the meeting, and for the excellent speech he made. There was not a word wasted in it, nor misplaced; it exactly set forth the objects of the League. Their duty was, as the Grand President said, to keep up the machinery of the League. Some had good luck in their districts, others had ill luck; he had lost his principal Vice-President, who had done wonderful work for him in Marylebone. He was taken suddenly ill and died. Prince Alexander had done most excellent work for the League, and he hoped his Serene Highness would long continue to do so.

### Sir George Alexander, LL.D., J.P., seconded. He thought that, following the theme dwelt upon by the reverend father, the Grand President must have a great deal of happiness. He desired to extend the resolution so as to include her Serene Highness Princess Alexander for her presence, and particularly as it was well known that the major part of the League's work was done by the ladies. He hoped that whatever legislation might come about, it would not affect those who remained willing to give their time and money to the League.

### Sir Henry Burdett desired to support the resolution, and took the opportunity of remedying an omission. The League had lost by death one of its warmest supporters, Sir Julius Wernher, who was also an old friend, so that he had been able to follow his life. Sir Julius began his life of personal service when a proposal was made to establish old-age pensions upon a contributory basis, in a scheme which the *Times* published and supported. That did not go forward, because it was held that these pensions must be compulsory. In that scheme were mentioned a labourer and his wife, and they happened to reside in the district where Sir Julius was then living. He felt with him (Sir Henry) that they ought to have a pension, which should be paid every week. Sir Julius twenty years ago undertook himself to pay that pension, and did so weekly during the whole of his life. Sir Julius, who died worth some six millions, believed in and practised personal service. He suffered terribly in his later days, and left a legacy of nearly half-a-million of money to the King's Fund. That should be an encouragement to workers in the League, and they should express their deep sympathy with Lady Wernher, who was one of the Lady Presidents.

The resolution of thanks to the Grand President was carried by acclamation, and the meeting terminated.

